# Evidence for *Msh2* haploinsufficiency in mice revealed by MNU-induced sister-chromatid exchange analysis

**DOI:** 10.1054/bjoc.2000.1422

**Published:** 2000-10-26

**Authors:** S D Bouffler, N Hofland, R Cox, R Fodde

**Affiliations:** Radiation Effects Department, National Radiological Protection Board, Chilton, Didcot, Oxfordshire, OX11 0RQ, UK; Department of Human and Clinical Genetics, Leiden University Medical Center, PO Box 9503, Leiden, RA, 2300, The Netherlands

**Keywords:** *Msh2*, mismatch repair, alkylating agent, ionizing radiation, sister-chromatid exchange

## Abstract

The role of *Msh2* in chromosome stability has been investigated in a targeted mouse model for HNPCC *Msh2Δ7N*. Chromosome aberration frequencies were similar in bone marrow of *Msh2*^+/+^*Msh2*^+/–^and *Msh2*^–/–^mice and no differential effects of in vivo X-irradiation were noted. By contrast, the induction of sister-chromatid exchanges (SCEs) by methyl nitrosourea (MNU) was reduced in *Msh2*^–/–^and *Msh2*^+/–^cells to ~20% and ~45% wild-type levels respectively indicating a phenotypic effect of haploinsufficiency of the mouse *Msh2* gene. © 2000 CancerResearch Campaign


*British Journal of Cancer* (2000) **83**(10), 1291–1294
© 2000 Cancer Research Campaign
doi: 10.1054/ bjoc.2000.1422, available online at http://www.idealibrary.com on IDEAL®

Short Communication

### Evidence for *Msh2* haploinsufficiency in mice revealed by MNU-induced sister-chromatid exchange analysis

**SD Bouffler[1], N Hofland[2], R Cox[1] and R Fodde[2]**

[1]Radiation Effects Department, National Radiological Protection Board, Chilton, Didcot, Oxfordshire, OX11 0RQ, UK; [2]Department of Human and Clinical Genetics, Leiden University Medical Center, PO Box 9503, 2300 RA Leiden, The Netherlands

**Summary** The role of *Msh2* in chromosome stability has been investigated in a targeted mouse model for HNPCC, *Msh2Δ7N*. Chromosome aberration frequencies were similar in bone marrow of $Msh2^{+/+}$, $Msh2^{+/-}$ and $Msh2^{-/-}$ mice and no differential effects of in vivo X-irradiation were noted. By contrast, the induction of sister-chromatid exchanges (SCEs) by methyl nitrosourea (MNU) was reduced in $Msh2^{-/-}$ and $Msh2^{+/-}$ cells to ~20% and ~45% wild-type levels respectively indicating a phenotypic effect of haploinsufficiency of the mouse *Msh2* gene.
© 2000 Cancer Research Campaign

**Keywords** *Msh2*; mismatch repair; alkylating agent; ionizing radiation; sister-chromatid exchange

Human individuals carrying a mutation in the mismatch repair gene *MSH2* are at increased risk of developing tumours in the colon and endometrium (Leach et al, 1993; Kolodner et al, 1994). Tumours which develop in these patients display microsatellite instability (MSI) characterized by mutations at microsatellite and other simple sequence repeat loci (Aaltonen et al, 1993; Ionov et al, 1993 Boland et al, 1998). The majority of these tumours have no active MSH2 protein and the elevated mutation frequency is a consequence of non-functional mismatch repair (Leach et al, 1993; Parsons et al, 1993). Mutations in a number of other mismatch repair genes give rise to similar phenotypes. Germline mutations in such mismatch repair genes are found in patients affected by HNPCC (hereditary non-polyposis colorectal cancer – reviewed by Modrich and Lahue, 1996; Kolodner, 1996). The underlying cause of the increased tumour risk in HNPCC patients remains a point of some controversy, in most cases mutation or loss of the remaining wild type (WT) mismatch repair gene allele appears to be required. The more difficult issue is whether somatic inactivation of the WT allele occurs at a 'normal' frequency or whether mutation/loss frequencies are elevated due to *Msh2* (or other mismatch repair gene) haploinsufficiency. In the first case, the excess risk is simply a consequence of the greater probability of mutation of one functional allele rather than two; in the second case a further enhancement due to cells with a single functional allele being somewhat genetically unstable (albeit to a lesser extent than in doubly mutant cells) is envisaged. Haploinsufficiency of a number of cancer-related genes has now been reported including *Atm* (Barlow et al, 1999), *Trp53* (Bouffler et al, 1995; Venkatachalam et al, 1998), TGFβ (Tang et al, 1998) and p27 (Fero et al, 1998).

In order to address this and related questions, a series of experiments employing *Msh2*-deficient mice was carried out. $Msh2^{-/-}$ mice develop lymphomas at high frequency and also tumours of the small bowel at lower incidence (deWind et al, 1995, 1998; Reitmair et al, 1995, 1996). Survival of $Msh2^{+/-}$ mice is comparable to that of wild type animals although some evidence suggests that tumours contribute disproportionately to the mortality of heterozygotes (deWind et al, 1998). However, loss/mutation of the wild type *Msh2* allele and MSI, as seen in HNPCC tumours, is relatively rare in tumours developing in heterozygous mice (deWind et al, 1998). The $Msh2^{\Delta7N}$ mouse model employed in this study carries a targeted deletion of *Msh2* exon 7 resulting in a null allele (Smits et al, in press). The incidence of lymphomas and small bowel tumours observed in homozygous and heterozygous mice closely resembles those reported for previously described models (de Wind et al, 1995, 1998).

*Received 5 January 2000*
*Revised 22 June 2000*
*Accepted 26 June 2000*

Correspondence to: SD Bouffler

### MATERIALS AND METHODS

Animals were bred and housed in Leiden University Medical Center. All animal procedures were conducted according to appropriate local Leiden University and Dutch national ethics guidelines and legislation. Spontaneous and X-ray induced chromosomal aberrations were assessed in direct bone marrow metaphase preparations of mutants and wild types. Age matched mice of each genotype were irradiated with an acute dose of 3 Gy X-rays. Direct bone marrow metaphases were made as described (Bouffler et al, 1995) from irradiated mice 1 month after exposure and age matched control, untreated mice. All samples were scored blind, the code being kept in Leiden while scoring was carried out at NRPB. Preparations were examined after block staining, G-banding or chromosome painting as described (Bouffler et al, 1996, 1997). G-banded slides were analysed using Chantal software (Leica Microsystems Imaging Solutions Ltd). Sister-chromatid exchange frequencies were determined in mitogen-stimulated spleen cultures, prepared as described (Bouffler et al, 1995). Methyl nitrosourea (Sigma) was initially diluted in dimethyl sulfoxide and further diluted in PBS immediately prior to adding to cultures 24 hours prior to harvesting.

**Table 1** Spontaneous and X-ray induced stable chromosome aberrations assessed by painting of chromosomes 1 and 2

| Genotype | X-ray dose/ sample time | Cells scored | Junctions/ 100 cells[a] |
|---|---|---|---|
| + + | 0 Gy | 159 | 0 |
| | 3 Gy/1 month | 250 | 18 |
| + – | 0 Gy | 102 | 0.98 |
| | 3 Gy/1 month | 297 | 14.5 |
| – – | 0 Gy | 41 | 0 |
| | 3 Gy/1 month | 219 | 16.9 |

[a]Frequency of junctions between painted and unpainted chromosome fragments per 100 cells. In this experiment the majority of junctions are due to translocations, some insertions, dicentrics, Robertsonian translocations and two coloured fragments were also seen.

**Table 2** G-band analysis of chromosomal aberrations in wild type and *Msh2* deficient mouse bone marrow cells 1 month following 3 Gy X-irradiation in vivo

| | Cells scored | Aberrations[a] | | | | | | Breakpoints per cell[b] |
|---|---|---|---|---|---|---|---|---|
| Genotype | | RT | TT | TD | ID | INS | Rb | |
| ++ | 18 | 8 | 0 | 0 | 4 | 3 | 0 | 1.8 |
| +– | 18 | 2 | 1 | 1 | 9 | 3 | 0 | 1.8 |
| –– | 24 | 4 | 1 | 2 | 7 | 1 | 1 | 1.3 |

[a]RT – reciprocal translocation, TT – terminal translocation, TD – terminal deletion, ID – interstitial deletion, INS – insertion, Rb – Robertsonian translocation.
[b]Calculated scoring 1 breakpoint for TT, TD and Rb, 2 breakpoints for RT and ID, 3 breakpoints for INS.

### RESULTS AND DISCUSSION

The gross morphology and number of chromosomes, assessed in block stained preparations, was normal in all three genotypes. Some indications of elevated frequencies of minute chromosome fragments in bone marrow cells were obtained. However, given the difficulty in scoring such chromosomal events, further analysis was not carried out. Painting of chromosomes 1 and 2 did not reveal any evidence of spontaneous chromosomal instability in any genotype (Table 1). Thus, the MSI phenotype does not appear to lead to elevated frequencies of spontaneous translocations. Some evidence in the literature suggests that *Msh2*-deficient cells have a somewhat elevated sensitivity to ionizing radiation (Fritzell et al, 1997; deWeese et al, 1998) and this sensitivity extends to elevated mutability at the *HPRT* locus after low dose rate exposure (deWeese et al, 1998). Thus, the in vivo induction of chromosome aberrations by X-irradiation was examined. Sampling at one month post-irradiation provides a reasonably accurate picture of induced stable, persistent aberrations (Bouffler et al, 1995, 1996). Chromosome painting indicated that the yield of exchange-type aberrations giving rise to colour junctions (e.g. translocations and insertions) was comparable in the three *Msh2* genotypes (Table 1). Chi-squared tests revealed no significant differences other than the effect of radiation elevating aberration yields. As chromosome painting analysis is not well suited to the detection of aberrations such as deletions and inversions, metaphases were also examined after G-banding. G-banding has the additional advantage of examining the entire genome. These data (Table 2) also indicated that all genotypes reacted similarly to X-irradiation, no statistically significant differences between genotypes were observed. Note that the frequency of breakpoints per cell assessed by G-banding is higher than the junctions per 100 cells scored by painting as G-banding scores over the entire genome while painting scored only chromosomes 1 and 2 (approximately 14% of the genome). Bearing this proportion in mind, the frequencies of aberrations scored by the two methods are broadly comparable.

These findings of no excessive spontaneous or induced chromosomal aberration formation are reflected in the observations of others that tumours arising in a background of mismatch repair deficiency infrequently show gross chromosomal changes (Aaltonen et al, 1993; Lengauer et al, 1997; Eshleman et al, 1998). The data presented here demonstrate clearly, however, that aberrations of the types associated with tumorigenesis (translocations, deletions, etc.) can be formed in the absence of mismatch repair, i.e. intact mismatch repair systems are not required for aberration formation. This suggests that in mismatch repair deficient cells, mutations in genes promoting proliferation and neoplastic development tend to take the form of sub-microscopic events principally, perhaps, DNA base pair changes.

Sister chromatid exchanges (SCEs) are a highly sensitive indicator of DNA damage induced by specific agents. Defects in mismatch repair are associated with reduced cellular sensitivity to and SCE induction by $0^6$-guanine-methylating agents such as methyl nitrosourea (MNU) (reviewed by Karran and Bignami, 1994). Assessment of SCE in splenocytes from *Msh2*$^{+/\Delta 7N}$, *Msh2*$^{\Delta 7N/\Delta 7N}$ and wild type littermates revealed no effect of the mutation on baseline frequencies (Fig. 1). SCE induction was strongly reduced in *Msh2*$^{-/-}$ splenocytes (Fig. 1), and to a lesser extent in *Msh2* heterozygotes. Thus, *Msh2* shows haploinsufficiency in this assay. Western analysis does not detect a difference in *Msh2* protein level between wild type and heterozygote ES cells (RF, unpublished observation) most probably due to limitations of sensitivity. MNU-induced SCEs are generally attributed to persistent DNA breaks caused by ‘futile repair’ opposite $O^6$-methylguanine residues by mismatch repair enzymes (see Karran and Bignami, 1994; Kaina et al, 1997). These persistent lesions contribute to cytotoxicity and SCE formation. In the absence of mismatch repair (*Msh2*$^{-/-}$ cells) few persistent lesions are produced. Hence, mismatch repair defective cells are resistant to the cell killing effects of $O^6$-guanine methylating agents (Karran and Bignami, 1994; deWind et al, 1995). The remaining alkyl lesions promote mutation (Andrew et al, 1998; Toft et al, 1999; Reitmair et al, 1997). However, elevated mutation frequencies have not been observed in *Msh2*$^{+/-}$ cells (Reitmair et al, 1997; Andrew et al, 1998; Toft et al, 1999). By contrast, the reduction in MNU-induced SCE observed here in *Msh2*$^{+/-}$ cells (Fig. 1), suggests that mismatch repair is compromised by *Msh2* haploinsufficiency. The apparent discrepancy between these data and the mutation studies may be reconciled by the observation that *Msh2* also plays a role in the signalling of apoptosis (Humbert et al, 1999; Toft et al, 1999). Haploinsufficiency of *Msh2* may exert a negative effect on its repair function but not on its capacity to signal apoptosis. Therefore, *Msh2*$^{+/-}$ cells with low induced SCE frequencies (and so, by implication, elevated $O^6$-methyl guanine burdens) are successfully channelled into apoptosis prior to mutations being fixed. *Msh2*$^{+/-}$ ES cells appear as sensitive to MNNG as wild-type cells (deWind et al, 1995) indicating that apoptosis is functional in heterozygotes. Thus, while *Msh2*

*© 2000 Cancer Research Campaign*

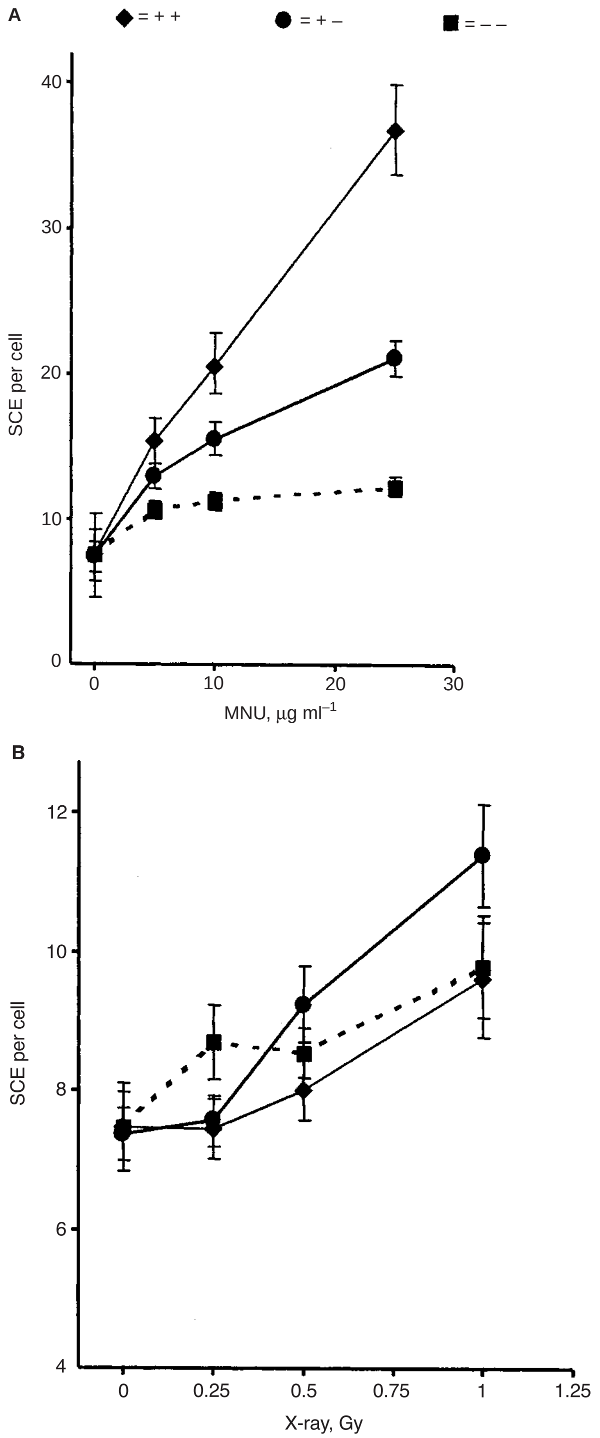

**Figure 1** Sister chromatid exchange in wild type (diamonds) and *Msh2*$^{+/-}$ (solid circles) and *Msh2*$^{-/-}$ (open circles) splenocytes induced by MNU (**A**) and X-rays (**B**)

haploinsufficiency can be effectively measured by the MNU-induced SCE assay, there would appear to be no reason to expect this to contribute to mutagenesis or carcinogenesis in the mouse.

Effects of *Msh2* in the heterozygous state have been observed following low dose rate ionizing radiation exposure. deWeese et al. (1998) found *Msh2*$^{+/-}$ cells to have a greater resistance to low dose rate radiation, and showed indications of elevated mutation frequencies. As with X-ray-induced chromosomal aberrations (Tables 1 and 2), in the present studies, no effect of *Msh2* deficiency was observed using SCEs as an endpoint (Fig. 1). The high dose rate employed here may be masking effects which would otherwise be seen with low dose rates. Furthermore, X-rays are only a weak inducer of SCEs despite being a potent carcinogen. The latter also underscores the lack of correlation between SCE induction and cancer.

In summary, it is reported here that haploinsufficiency of *Msh2* in mice can be revealed by MNU-induced SCE analysis. No phenotypic effect of loss of one or two *Msh2* alleles was detected with regard to spontaneous or X-ray induced structural chromosome aberrations. The data presented here add to the evidence accumulating which indicates that haploinsufficiency for some cancer related genes, including *Msh2*, has detectable phenotypic effects.

We thank Neils de Wind for critical reading of the manuscript, Kathy Brooks for typing the manuscript, John Moody for preparing the figure and Paul Bonner of the MRC Radiation and Genome Stability Unit for irradiation of splenocyte cultures.

### REFERENCES

Aaltonen LA, Peltomaki P, Leach FS, Sistonen P, Pylkkänen L, Mecklin JP, Jartinen H, Powell SM, Jen J, Hamilton SR, Petersen GM, Kinzler KW, Vogelstein B and de la Chapelle A (1993) Clues to the pathogenesis of familial colorectal cancer. *Science* **260**: 812–816

Andrew SE, McKinnon M, Cheng BS, Francis A, Penney J, Reitmair AH, Mak TW and Jirik FR (1998) Tissues of MSH2-deficient mice demonstrate hypermutability on exposure to a DNA methylating agent. *Proc Natl Acad Sci USA* **95**: 1126–1130

Barlow C, Eckhaus MA, Schaffer AA and Wynshaw-Boris A (1999) *Atm* haploinsufficiency results in increased sensitivity to sublethal doses of ionizing radiation in mice. *Nature Genet* **21**: 359–360

Boland CR, Thibodeau SN, Hamilton SR, Sidransky D, Eshleman JR, Burt JR, Meltzer SJ, Rodriguez-Bigas MA, Fodde R, Ranzani GN and Srivastava S (1998) A National Cancer Institute Workshop on Microsatellite Instability for Cancer Detection and Familial Predisposition: Development of International Criteria for the Determination of Microsatellite Instability in Colorectal Cancer. *Cancer Res* **58**: 5248–5257

Bouffler SD, Kemp CJ, Balmain A and Cox R (1995) Spontaneous and ionizing radiation-induced chromosomal abnormalities in *p53*-deficient mice. *Cancer Res* **55**: 3883–3889

Bouffler SD, Breckon G and Cox R (1996) Chromosomal mechanisms in murine radiation acute myeloid leukaemogenesis. *Carcinogenesis* **17**: 655–659

Bouffler SD, Meijne EIM, Morris DJ and Papworth D (1997) Chromosome 2 hypersensitivity and clonal development in murine radiation acute myeloid leukaemia. *Int J Radiat Biol* **72**: 181–189

deWeese TL, Shipman JM, Larrier NA, Buckley NM, Kidd LR, Groopman JD, Cutler RG, teRiele H and Nelson WG (1998) Mouse embryonic stem cells carrying one or two defective *Msh2* alleles respond abnormally to oxidative stress inflicted by low-level radiation. *Proc Natl Acad Sci USA* **95**: 11915–11920

deWind N, Dekker M, Berns A, Radman M and teRiele H (1995) Inactivation of the mouse *Msh2* gene results in mismatch repair deficiency, methylation tolerance, hyperecombination and predisposition to cancer. *Cell* **82**: 321–330

deWind N, Dekker M, van Rossum A, van der Valk M and teRiele H (1998) Mouse models for hereditary nonpolyposis colorectal cancer. *Cancer Res* **58**: 248–255

Drotschmann K, Clark AB, Tran HT, Resnick MA, Gordenin DA and Kunkel TA (1999) Mutator phenotypes of yeast strains heterozygous for mutations in the *MSH2* gene. *Proc Natl Acad Sci USA* **96**: 2970–2975

Eshleman JR, Casey G, Kochera ME, Sedwick WD, Swinler SE, Veigl ML, Willson JKV, Schwartz S and Markowitz SD (1998) Chromosome number and structure both are markedly stable in RER colorectal cancers and are not destabilized by mutation of p53. *Oncogene* **17**: 719–725

Fero ML, Randel E, Gurley KE, Roberts JM and Kemp CJ (1998) The murine gene p27$^{Kip1}$ is haplo-insufficient for tumour suppression. *Nature* **396**: 177–180

© 2000 Cancer Research Campaign

Fritzell JA, Narayanan L, Baker SM, Bronner CE, Andrew SE, Prolla TA, Bradley A, Jirik FR, Liskay RM and Glazer PM (1997) Role of DNA mismatch repair in the cytoxicity of ionizing radiation. *Cancer Res* **57**: 5143–5147

Humbert O, Fiumicino S, Aquilina G, Branch P, Oda S, Zijno A, Karran P and Bignami M (1999) Mismatch repair and differential sensitivity of mouse and human cells to methylating agents. *Carcinogenesis* **20**: 205–214

Ionov Y, Peinado MA, Malkhosyan S, Shibata D and Perucho M (1993) Ubiquitous somatic mutations in simple repeated sequences reveal a new mechanism for colonic carcinogenesis. *Nature* **363**: 558–561

Kaina B, Ziouta A, Ochs K and Coquerelle T (1997) Chromosome instability, reproductive cell death and apoptosis induced by $O^6$-methylguanine in Mex–, Mex+ and methylation tolerant mismatch repair compromised cells: facts and models. *Mutation Res* **381**: 227–241

Karran P and Bignami M (1994) DNA damage tolerance, mismatch repair and genome instability. *BioEssays* **16**: 833–839

Kolodner RD (1996) Biochemistry and genetics of eukaryotic mismatch repair. *Genes Dev* **10**: 1433–1442

Kolodner RD, Hull NR, Lipford J, Kane MF, Rao MRS, Morrison P, Wirth L, Finan PJ, Burn J, Chapman P, Earabino C, Merchant E and Bishop DT (1994) Structure of the human *MSH2* locus and analysis of two Muir-Torre kindreds for *msh2* mutations. *Genomics*, **24**: 516–526

Leach FS, Nicolades NC, Papadopoulos N, Liu B, Jen J, Parsons R, Peltomäki P, Sistonen P, Aaltonen LA, Nyström-Lathi M, Guan X-Y, Zang L, Meltzer PS, Yu J-W, Kao F-T, Chen DJ, Cerosaletti KM, Fournier REK, Todd S, Lewis T, Leach RJ, Naylor SL, Weissenbach J, Mecklin J-P, Jarvinen H, Peterson GM, Hamilton SR, Green J, Jass J, Watson P, Lynch HT, Trent JM, de la Chapelle A, Kinzler KW and Vogelstein B (1993) Mutations of a *mutS* homologue in hereditary nonpolyposis colorectal cancer. *Cell* **75**: 1215–1225

Lengauer C, Kinzler KW and Vogelstein B (1997) Genetic instability in colorectal cancers. *Nature* **386**: 623–627

Modrich P and Lahue RS (1996) Mismatch repair in replication fidelity, genetic recombination and cancer biology. *Ann Rev Biochem* **65**: 101–133

Parsons R, Li G-M, Longley MJ, Fang W, Papadopoulos N, Jen J, de la Chapelle A, Kinzler KW, Vogelstein B and Modrich P (1993) Hypermutability and mismatch repair deficiency in $RER^+$ tumor cells. *Cell* **75**: 1227–1236

Reitmair AH, Schmits R, Ewel A, Bapat B, Redston M, Mitri A, Waterhouse P, Mittrücker HW, Wakeham A, Liu B, Thomason A, Griesser H, Gallinger S, Ballhausen WG, Fishel R and Mak TW (1995) MSH2 deficient mice are viable and susceptible to lymphoid tumours. *Nature Genet* **11**: 64–70

Reitmair AH, Redston M, Cai JC, Chuang TCY, Bjerknes M, Cheng H, Hay K, Gallinger S, Baput B and Mak TW (1996) Spontaneous intestinal carcinomas and skin neoplasms in *Msh2*-deficient mice. *Cancer Res* **56**: 3842–3849

Reitmair AH, Risley R, Bristow RG, Wilson T, Ganesh A, Jang A, Peacock J, Benchimol S, Hill RP, Mak TW, Fishel R and Meuth M (1997) Mutator phenotype in Msh2-deficient murine embryonic fibroblasts. *Cancer Res* **57**: 3765–3771

Tang B, Büottinger EP, Jakowlew SB, Bagnall KM, Mariano J, Anver MR, Letteriu JJ and Wakefield LM (1998) Transforming growth factor-beta 1 is a new form of tumor suppressor with true haploid insufficiency. *Nature Med* **4**: 802–807

Toft NJ, Winton DJ, Kelly J, Howard LA, Dekker M, teRiele H, Arends MJ, Wyllie AH, Margison GP and Clarke AR (1999) Msh2 status modulates both apoptosis and mutation frequency in the murine small intestine. *Proc Natl Acad Sci USA* **96**: 3911–3915

Venkatachalam S, Shi YP, Jones SN, Vogel H, Bradley A, Pinkel D and Donehower LA (1998) Retention of wild-type p53 in tumors from p53 heterozygous mice: reduction of p53 dosage can promote cancer formation. *EMBO J* **17**: 4657–4667

*© 2000 Cancer Research Campaign*

